# Women’s Experience Obtaining Abortion Care in Texas after Implementation of Restrictive Abortion Laws: A Qualitative Study

**DOI:** 10.1371/journal.pone.0165048

**Published:** 2016-10-26

**Authors:** Sarah E. Baum, Kari White, Kristine Hopkins, Joseph E. Potter, Daniel Grossman

**Affiliations:** 1 Texas Policy Evaluation Project, Austin, Texas, United States of America; 2 Ibis Reproductive Health, Oakland, California, United States of America; 3 Department of Health Care Organization and Policy, School of Public Health, University of Alabama at Birmingham, Birmingham, Alabama, United States of America; 4 Population Research Center, University of Texas at Austin, Austin, Texas, United States of America; 5 Advancing New Standards in Reproductive Health (ANSIRH), Bixby Center for Global Reproductive Health, Department of Obstetrics, Gynecology and Reproductive Sciences, University of California San Francisco, Oakland, California, United States of America; NHS lothian and University of Edinburgh, UNITED KINGDOM

## Abstract

**Background:**

In November 2013, Texas implemented three abortion restrictions included in House Bill 2 (HB 2). Within six months, the number of facilities providing abortion decreased by almost half, and the remaining facilities were concentrated in large urban centers. The number of medication abortions decreased by 70% compared to the same period one year prior due to restrictions on this method imposed by HB 2. The purpose of this study was to explore qualitatively the experiences of women who were most affected by the law: those who had to travel farther to reach a facility and those desiring medication abortion.

**Methods:**

In August and September 2014, we conducted 20 in-depth interviews with women recruited from ten abortion clinics across Texas. The purposive sample included women who obtained or strongly preferred medication abortion or traveled ≥50 miles one way to the clinic. The interview guide focused on women’s experiences with obtaining services following implementation of HB 2, and a thematic analysis was performed.

**Results:**

Women faced informational, cost and logistical barriers seeking abortion services, and these obstacles were often compounded by poverty. Two women found the process of finding or getting to a clinic so onerous that they considered not having the procedure, although they ultimately had an abortion; another woman decided to continue her pregnancy, in part because of challenges in getting to the clinic. For two women, arranging travel required disclosure to more people than desired. Women who strongly preferred medication abortion were frustrated by the difficulty or inability to obtain their desired method, especially among those who were near or just beyond the gestational age limit. The restricted eligibility criteria for medication abortion and difficulty finding clinics offering the method created substantial access barriers.

**Conclusions:**

Medication abortion restrictions and clinic closures following HB 2 created substantial barriers for women seeking abortion in Texas.

## Introduction

Approximately 56 million abortions occur worldwide annually, corresponding to a rate of 35 abortions per 1000 women aged 15–44 years [1]. Although abortion is common, many countries impose legal restrictions on the procedure. For example, in about 58 countries, abortion is completely illegal or allowed only to save the pregnant woman’s life [1]. In the United States, despite legalization of abortion in 1972, policy restrictions at the state level have imposed barriers to women’s access to care, including laws that restrict how abortion is provided, known as Targeted Regulations of Abortion Providers (TRAP) laws. In recent years, the number of TRAP laws has increased substantially in the US [2]. Examples of TRAP laws include regulations on the type of facility that can provide abortion care or requirements that providers have a relationship with a nearby hospital, including admitting privileges.

In July 2013, the Texas legislature passed House Bill 2 (HB 2), one of the United States’ most restrictive TRAP laws. By November 1, 2013, three components of the law had been implemented: physicians were required to have admitting privileges at a hospital within 30 miles of the facility providing abortion; administration of medication abortion was restricted to the protocol described in the mifepristone label approved by the US Food and Drug Administration (FDA) in 2000, with some allowances for medication dosages; and abortions after 20 weeks “post fertilization” were prohibited in most cases. The fourth component of HB 2, which required all abortion facilities to meet the standards of ambulatory surgical centers (ASCs), went into effect briefly in October 2014 before the US Supreme Court issued a stay temporarily blocking enforcement of this portion of the law. In June 2016, the Supreme Court ruled that the admitting privileges and ASC requirements of HB 2 were unconstitutional because they placed an undue burden on women’s access to abortion without offering any health or safety benefit [3].

Between April and November 2013, when the first three provisions of HB 2 had been enforced, the number of abortion facilities providing care in Texas fell from 41 to 22 [4]. Clinic closures resulted in an increased number of Texas women of reproductive age living farther from an open facility, meaning that more women had to travel longer distances in order to obtain abortion care. In April 2014, 1,680,000 Texas women aged 15–44 (31% of all Texas women of reproductive age) lived more than 50 miles from an open facility, compared to 816,000 women (15%) one year prior [4]. In comparison, a nationally representative study conducted in 2008 found that 17% of abortion patients traveled over 50 miles each way to the facility [5].

In addition to clinic closures, there were substantial shifts in the use of medication abortion after HB 2. Medication abortion, a procedure which involves the use of two medications (mifepristone and misoprostol) is generally performed in the US up to nine or ten weeks’ gestation [6], although the medications are safe and effective later in gestation as well [7, 8]. National data indicate there has been a steady increase in the proportion of non-hospital abortions performed with medication, from 17% in 2008 to 23% in 2011, representing approximately 36% of all abortions up to nine weeks’ gestation [9]. In the six months following implementation of HB 2, a 70% decline in the number of medication abortions in Texas was observed, as compared to the same period one year prior [4]. A steep drop in the proportion of abortions performed with medication was also observed in Ohio after a similar law was implemented [10].

The observed decline in the proportion of medication abortions in Texas may be attributed to three parts of the new regulations. First, because HB 2 restricted provision of medication abortion to pregnancies at or below seven weeks’ gestation, fewer women were eligible for the method. Second, HB 2 imposed limitations on the dose of mifepristone and the medication regimen. Either providers had to use a higher dose of mifepristone than that recommended by current evidence (600 mg vs. 200 mg), which increased the cost of the service, or they had to use a regimen (mifepristone 200 mg followed by 800 mcg of misoprostol administered orally), that has not been well studied [6, 11]. Third, due to both HB 2 and previously enforced abortion restrictions in Texas, most women who obtained medication abortion were required to make four visits to the facility: 1) a state-mandated pre-abortion counseling and ultrasound visit at least 24 hours before the procedure if she lived less than 100 miles from the nearest clinic, 2) a visit to take the mifepristone observed by the provider, 3) a visit to take the misoprostol observed by the provider, and 4) a visit to confirm abortion completion. Given the challenges to provision, some facilities stopped offering medication abortion altogether after HB 2 went into effect. The more limited availability of the method at clinics, compounded by clinic closures, the prospect of four visits, and higher costs for the procedure made it harder for women seeking medication abortion to obtain one [12, 13]. Of note, in March 2016, the FDA updated the mifepristone label to allow for use up to 10 weeks’ gestation using a reduced dosage of the medication and home administration of misoprostol [14], which has allowed Texas providers to once again use this evidence-based protocol.

Texas provides a good setting within which to assess the impact of multiple clinic-based restrictions imposed simultaneously. Two prior studies focused on Texas suggest that TRAP laws can result in a significant decline in the rate of abortions performed after implementation [4, 15]. TRAP laws may impose greater obstacles to women’s access to abortion care compared to patient-focused restrictions such as mandated counseling and ultrasound requirements [9, 16]. These laws threaten women’s access to safe legal abortion; however, there is limited research addressing the experiences of women following the implementation of TRAP laws. Given the numerous clinic closures, the reduced geographic distribution of providers and significant decrease in medication abortion in the year following implementation of HB 2, this qualitative study aimed to document the experiences of seeking and obtaining abortion care among women who were most likely to be affected by the law: those who had to travel farther to reach a facility and those who desired medication abortion.

## Materials and Methods

### Sample & recruitment

We conducted semi-structured in-depth interviews with women in Texas about their experiences seeking and obtaining abortion services after the first three provisions of HB 2 went into effect. Women were recruited for the interviews from among 439 participants in a cross-sectional survey in Texas that aimed to assess barriers accessing care in the new regulatory environment [13]. Survey participants were recruited between May and August 2014 at ten abortion clinics in Austin, Dallas, Fort Worth, Houston, and San Antonio; they completed an electronic survey on an iPad in clinic waiting rooms. Eligibility criteria for the survey included ability to speak English or Spanish, being 18 years of age or older, and having completed the initial visit with ultrasound and counseling [13].

Immediately following completion of the survey, the study coordinator invited all participants to complete a follow-up telephone interview. If interested, participants provided contact information on an electronic form. Women were then purposively sampled based on one or more of the following reported characteristics: obtained a medication abortion, reported that they “strongly wanted” or were “leaning toward” a medication abortion prior to obtaining care, or traveled 50 miles or more one way to the clinic. We sampled based on these characteristics in order to conduct interviews with women who were most affected by HB 2, including those who were seeking or obtained medication abortion and those who traveled farther than the mean distance (46 miles one way) reported in the survey [13].

### Data collection

The semi-structured in-depth interview guide included open-ended questions about women’s experience with and opinions about the process of obtaining abortion services following implementation of HB 2. We developed the interview guide based on a previous study with abortion clients in Texas [17], as well as on themes that emerged from open-ended questions in the survey from which the current sample was drawn, such as why it was difficult to get to the clinic or why women preferred their desired abortion method. Interview topics included: experience finding a clinic and scheduling an appointment; logistical factors considered in getting to the clinic(s); travel experience, including multiple visits for medication abortion; preferences for and decision making around abortion method; abortion experience; and opinion on Texas abortion laws. Interviewers collected participant demographic information at the end of the interview. Copies of the research instrument are available upon request.

There were 161 women who provided contact information to participate in an in-depth interview after completing the survey, and 50 of those women met our inclusion criteria. Women were re-contacted one to two months after survey completion. After reaching out to all eligible women by telephone a maximum of three times, we completed 20 interviews. Interviews were conducted by two interviewers with training in qualitative methods and experience conducting research on access to abortion care in US and global settings. Immediately before the interview, the interviewer discussed the aims of the study with participants, reviewed the informed consent and answered participants’ questions. Women provided verbal consent in order to reduce the risk of loss of confidentiality and because the interviews were conducted by telephone; their consent was documented by the interviewers. Participants also provided permission to digitally record the interview, which was identified only by study ID. Interviews were completed by telephone in English and lasted between 20–50 minutes. Women received a $30 gift card by mail for their participation. The study was approved by the Institutional Review Board at the University of Texas at Austin.

### Data analysis

Qualitative interviews were transcribed and coded using Atlas.ti 6.2 (Atlas.ti GmbH, Berlin, Germany). The research team developed a codebook using *a priori* codes, as well as codes that emerged naturally from the interviews. The codebook and subsequent analysis were guided by an access to care framework that highlights facilitators and barriers to care based on the interaction between health service-delivery factors, such as availability and affordability of services, and user factors, such as demand for care, utilization and acceptability [18, 19]. Two qualitative researchers coded two transcripts separately with the initial codebook and met to discuss discrepancies and revise the list of codes. Next, three researchers each coded one third of the transcripts, wrote analytic memos to document observations, and participated in intermittent meetings to discuss emergent themes, add or collapse codes and reach consensus on coding disagreements. The research team conducted a thematic analysis to assess patterns of experiences and opinions across themes and reached agreement of interpretation. Illustrative quotes were selected for salient themes and are presented with participants identified by participant number, age, abortion method obtained, recruitment city, and miles traveled one way to the clinic.

## Results

### Participant characteristics

Interview participants ranged from the ages of 19–34 years. Eight women identified as White, five as African American, five as Hispanic, one as Asian American, and one as more than one race. At the time of the interview, 12 women were single, three were living with partners but unmarried, and five were married. The highest education level they completed ranged from incomplete high school to some college or technical school; twelve women were currently in school. Ten women had children, six women reported a previous abortion (Table 1).

**Table 1:** Participant characteristics.

Participant	Recruitment City	Age (years)	Race/ Ethnicity	Highest level of education completed	Relationship Status	Previous abortion	Gestational age at ultrasound (weeks)	Pregnancy outcome	Abortion Method	Distance traveled (mi)*
1	Dallas/Ft Worth	26–29	Latina	Completed high school	Relationship; living together	No	≤7	Abortion	Medical	30
2	San Antonio	18–21	African American	Some college	Single	No	≤7	Abortion	Medical	70
3	San Antonio	30–34	White	Completed college	Married	No	≤7	Abortion	Medical	200
4	San Antonio	22–25	Indian and Latina	-**	-	-	≤7	Abortion	Medical	15
5	San Antonio	30–34	White	Some high school	-	Yes	8–9	Continued Pregnancy	N/A	85
6	Austin	26–29	White	Some college	Single	Yes	8–9	Abortion	Surgical	3
7	Dallas/Ft Worth	22–25	African American	Some college	Relationship; living together	-	≤7	Abortion	Surgical	50
8	Dallas/Ft Worth	18–21	African American	Some college	Single	No	8–9	Abortion	Surgical	16
9	Dallas/Ft Worth	22–25	White	Some college	Single	Yes	≤7	Abortion	Surgical	20
10	Houston	22–25	Latina	Some college	Married	No	8–9	Abortion	Surgical	15
11	Houston	30–34	Asian American	Completed college	Married	Yes	≤7	Abortion	Surgical	10
12	San Antonio	22–25	Latina	Completed high school	Single	No	10–12	Abortion	Surgical	180
13	San Antonio	18–21	Latina	Completed high school	Single	No	8–9	Abortion	Surgical	300
14	San Antonio	30–34	White	Completed high school	Single	No	≤7	Abortion	Surgical	123
15	Austin	18–21	African American	Some college	Single	No	>12	Abortion	Surgical	62
16	Austin	26–29	Latina	Completed high school	Married	Yes	≤7	Abortion	Surgical	15
17	Austin	22–25	White	Some college	Single	Yes	>12	Abortion	Surgical	50
18	Austin	18–21	White	Some college	Single	No	≤7	Abortion	Surgical	21
19	Austin	18–21	African American	Some college	Single	-	≤7	Abortion	Surgical	48
20	Austin	30–34	White	Completed high school	Married	No	≤7	Abortion	Surgical	50

*Miles traveled one way

** Missing data are represented by “-“

Eighteen women were in the first trimester of pregnancy (range 5–10 weeks’ gestation) at the time of their ultrasound, twelve of whom were ≤7 weeks and five between 8 and 10 weeks. Two women were seeking abortion in the second trimester (14 and 16 weeks’ gestation). Four women obtained medication abortion, 15 obtained surgical abortion, and one woman decided to continue her pregnancy. Eleven of the 15 who obtained a surgical abortion reported they strongly preferred a medication abortion prior to obtaining services. Ten women traveled 50 miles or more one way to the clinic, with an average of 117 miles and a range of 50–300 miles (Table 1).

Below, we discuss the main themes that emerged in women’s narratives about the implications of travel due to clinic closures and experiences seeking medication abortion in a restricted environment. We also explore women’s perception of the Texas abortion law.

### Implications of travel due to clinic closures

#### Difficulty finding and getting to an open clinic

Women found open facilities that offered abortion services through the internet, telephone hotlines, word of mouth, doctor referrals, and previous experience with family planning and abortion services. While a few women who lived in metropolitan areas reported that scheduling and getting to their appointments was relatively easy, the majority of women in this study had to make multiple calls because the clinics they initially contacted were closed or no longer offering abortion services. One woman spoke of her efforts to schedule an appointment before being referred to a facility more than 100 miles from her home:

I had gotten [the number for the clinic] from the Internet and I kept calling and calling and never got an answer. So I called [another clinic], and they’re the ones that told me that we no longer have [a clinic] in our town. And then [they] gave me the number for the closest one, which was in San Antonio. (34 years old, surgical abortion in San Antonio, traveled 123 miles)

Women described the stress of having to get to unfamiliar cities and coordinate transportation. Two women who ultimately obtained an abortion initially found the process of finding or getting to a clinic so onerous that they considered not having the procedure. One of these women said the process of finding a clinic made her hesitate about having the abortion at all:

It was a very hard thing to do, like to keep calling and calling and calling. I almost was like you know, well forget it. […] But then because I knew at the end of the day it was something that I had to do. Not necessarily had to, I had a choice, but it was like one outweighed the other and it was like I don’t care how many people I have to call or how far I have to go. I have to do it. (19 years old, medication abortion in San Antonio, traveled 70 miles)

In order to avoid the extra cost of staying in a hotel, she preferred making the drive to each of her three appointments for medication abortion in the company of her sister and friend. The other woman traveled 300 miles to reach a clinic, which was the longest distance traveled among women in our sample. After a positive pregnancy test at her local health center, she discovered that the two nearest abortion clinics had closed. She described her reaction to learning of the closest clinic:

The only time I thought about not doing it was in the beginning because, like I said, driving to San Antonio was–like wow, I have to drive six hours to do the procedure. I thought it was a little bit out of my league, like don’t do it, that’s too far. I don’t know. So it was a little stressful. But once I thought about it and I talked to the clinic, just getting all the information made me feel a little bit more like, okay, you can go through it. (21 years old, surgical abortion in San Antonio, traveled 300 miles)

Another woman, who later decided to continue her pregnancy, experienced similar logistical challenges getting to her ultrasound appointment. Her van broke down and her 85-mile trip to San Antonio required a combination of cab, city bus and Greyhound bus. Her travel costs increased when her appointment at the clinic lasted longer than anticipated and she missed her return bus. She described this travel as “a nightmare”.

[Getting to the clinic] took a toll on me and it was awful. It was raining that day, of all the days it’s raining […] I went to a place I don’t know, all these busses I don’t know. I had to take two buses just to get to that appointment. It was the longest day that I can remember having for a long time. (31 years old, continued pregnancy, had ultrasound in San Antonio, traveled 85 miles)

The clinic was not able to schedule her procedure appointment for at least three weeks. Feeling that she had the support of someone in her life with whom to share parenting, as well as concern about how far along she would be in her pregnancy by the time she could return to the clinic for the procedure, she decided not to return for the abortion.

#### Fear of unwanted disclosure

In some cases, the long travel distance or multiple appointments that were required put women in a position where they would have to discuss their abortion decision with more people than they wanted. For the woman who traveled 300 miles to San Antonio, in addition to quitting her seasonal job because of challenges negotiating enough time off, she described her fears about disclosure to her parents:

The doctor did tell me that there were clinics closing down already because they weren’t doing [abortions]. And I was just like well this is going to make it more difficult for me ‘cause then of course my parents are going to be like, “Where are you going, why are you going over there, who are you going with, what are you going to do?” It was just going to lift up so many other questions that I was trying to dodge and not wanting to get anybody involved. (21 years old, surgical abortion in San Antonio, traveled 300 miles)

A 30-year-old woman who traveled 50 miles for her surgical procedure, worried about disclosing her abortion to a babysitter, and ended up paying her sister to take care of her three children: “I had to find someone I had to confide in about the situation and let them know if they could watch the kids…I basically [had to] disclose information about the situation that [I didn’t] necessarily want to, I mean, that’s kind of–that’s disturbing.”

#### Difficulty with out-of-pocket costs associated with travel

Participants discussed various other challenges related to their travel experiences, including out-of-pocket costs for transportation and overnight stays. Most women were able to use their own car or that of family or friends to get to the clinic, but many had to ask for help paying for gas.

In order to avoid additional out-of-pocket costs for an overnight stay at a hotel between their ultrasound and procedure visits, most of the women traveling more than 50 miles made the round-trip drive to the clinic two or more times, and two women stayed with family; only one stayed overnight in a hotel. For the abortion procedure visit, all women needed to have a driver transport them, which often resulted in lost wages for their support people. One woman described how her costs multiplied because her husband also accompanied her on the 200 mile trip each way to the clinic:

It cost not only the money at the clinic but then the money that it cost my husband for taking off work a full day, then the gas and all that stuff and then going again and him taking another full day of work off. (31 years old, medication abortion in San Antonio, traveled 200 miles)

One 24-year-old woman, who was both working and studying, commented that the costs may not seem very high, but when they are calculated along with the clinic fee, it was very challenging:

I had this job and they really didn’t give me a lot of hours. I didn’t have a lot of money and my boyfriend doesn’t make a lot of money. I had to pay for the gas and I know maybe it’s only like 40, 50 dollars but that’s a lot when you’re not making that much. (24 years old, surgical abortion in Dallas/Ft Worth, traveled 50 miles)

### Seeking medication abortion in a restricted environment

#### Impact of medication abortion protocol changes

Several women were able to overcome the scheduling and travel challenges to obtain their wanted medication abortion. These women were generally knowledgeable about the gestational age limit and were persistent in finding an open clinic that offered medication abortion that they could reach prior to seven weeks of pregnancy. One of these women arrived at her appointment having researched the laws and insisted on obtaining medication abortion despite being close to the gestational age limit.

During my first visit when I was discussing the procedures, [the counselor] told me that I couldn’t take the pill. And I really did not want to do the vacuum [procedure] unless it absolutely came down to that. And so she left and she came back and that was when I remembered reading that you can do it before seven weeks. (26 years old, medication abortion in Dallas/Ft Worth, traveled 30 miles)

Another woman started calling clinics at six weeks gestation and contacted three facilities before she was able to make an appointment. Although she strongly preferred medication abortion over an aspiration procedure, she also anticipated the discomfort of traveling for over an hour on her return trip home after taking the misoprostol at the clinic as required by law:

They give you four pills…and then immediately after you swallow, that’s when you’ll have cramping and heavy bleeding and everything. So seeing as though I was commuting, it was about like an hour and 45 minutes from my house, they told you that you needed to have somebody to drive you back home because by then you’ll be cramping and bleeding and just be very uncomfortable for driving. (19 years old, medication abortion in San Antonio, traveled 70 miles)

#### Obtaining surgical abortion despite preferring medication

There were various reasons that women did not obtain their preferred medication abortion. Some women were ineligible because they were beyond the gestational age limit defined in the Texas law. While some of them were comfortable getting a surgical procedure, the change in eligibility was particularly upsetting for one woman who strongly preferred medication abortion based on her previous experience. But she was unable to obtain the method at the clinic closest to her:

I didn't realize until I called this last time to try and set up an appointment that I could no longer get the medication abortion and that it was surgical only, which was even more frightening and I didn't want to do it. They told me that due to the legislation passed last year, it was no longer available and they could offer the surgical procedure. I didn't want to do it. (26 years old; surgical abortion in Austin; traveled 3 miles)

Other women were eligible for the method under the lower gestational age limit but were unable or unwilling to travel to a clinic that offered it and obtained a surgical procedure instead. For one woman, the uncertainties about traveling to another clinic to obtain medication abortion led her to reluctantly opt for a surgical procedure closer to home at eight weeks gestation:

I considered [driving to Dallas, which is 200 miles away] just because it would be, you know, well I can just drive up there and take a pill. [But] I didn’t know if it would be more costly. I knew that the gas would be more costly. I didn’t know where [the clinic] was, honestly. (30 years old, surgical abortion in Austin, traveled 50 miles)

In these instances, women’s method of abortion did not feel like a choice. They were most disappointed when they learned that their desired procedure would not be available and realized they did not have the money or time to seek out another facility, as one woman explained:

Well, I actually found out when I went in for my test that they don’t offer [medication abortion]. I just started crying hysterically in front of the girl who was counseling me and she didn’t know what to do. It was like, “I could refer you somewhere else but, you know, you’re like three days away from the [cutoff] day,” and I didn’t have half the money I needed for it yet. (20 years old, surgical abortion in Austin, traveled 21 miles)

### Perception of Texas abortion laws

Women’s understanding of HB 2 varied. Many expressed confusion about parts of the law or were not sure if what they heard was true. A couple of women stated that prior to obtaining their procedure they doubted whether abortion was still legal in Texas. Women tended to be most familiar with the fact that clinics had closed as a result of the law. They expressed concerns about how this impacted access to services in the state:

Well, I heard that they were going to be taking more clinics away, that they were going to be trying to make it harder for women to get abortions. If someone isn’t ready to have a child then they shouldn’t have to be going to a different state or out of the way just to do what they have to do. I don’t feel like it’s right. (19 years old; surgical abortion in Austin; traveled 48 miles)

One woman described how this could potentially lead to increased unsafe abortion outside of clinic settings:

It’s not a good idea to shut down clinics, just because of the past. [Women] will find ways to get rid of the pregnancy and sometimes that can lead to death. You know, so to me, it’s actually scary. (24 years old; surgical abortion in Dallas/Ft Worth; traveled 20 miles)

When asked about their opinion on the abortion laws in Texas, women talked about the compounding barriers imposed by laws passed prior to HB 2, including the two-visit requirement and 24-hour waiting period passed in 2011. For example, a frustrated 19-year-old woman explained:

The first actual day, I felt like it was a waste of time because I had to drive all the way to San Antonio to sit and listen to a guy read off a piece of paper of, like, the Texas legislative laws. And that was a requirement. I’m like, that’s a long way to drive just to listen to somebody talk. (19 years old, medication abortion in San Antonio, traveled 70 miles)

Although one woman expressed support for the medication abortion gestational limit, there was a general consensus among the majority that the laws were creating additional and unnecessary barriers for women, as described by this woman:

I feel like they’re trying to make the women not get it ‘cause the price is so high and they know that they won’t be able to afford it. So they want to make them struggle and have more people in the poverty level and stuff. So then that means like more kids in the adoption clinics, more kids homeless and orphans and all that other stuff. So it’s like–it’s not even like a win-win situation. It’s like a lose-lose. (19 years old; surgical abortion in Dallas/Ft Worth; traveled 16 miles)

## Discussion

This qualitative study provides insight into women’s experiences trying to access abortion care in the face of the multiple abortion restrictions implemented as part of HB 2 in Texas. Previous research has demonstrated that immediately after HB 2 was enforced, women were confused about which clinics were open and some faced longer travel distances to reach clinics, which created obstacles to care [12]. The results from this study demonstrate that such barriers persisted more than six months after HB 2 was first enforced. Specifically, women who traveled 50 miles or more and women who preferred medication abortion at the time of seeking services continued to encounter informational barriers to find a clinic offering services and obtain their preferred method of abortion.

These findings also shed light on the extent and nature of the burdens faced by Texas women after the first provisions of HB 2 were implemented, which were documented in the survey from which participants in this study were selected. Data from that survey indicate that the 38% of abortion clients whose nearest clinic had closed between 2013 and 2014 were significantly more likely to have traveled farther for services, have higher out-of-pocket-costs and report it was difficult getting to the clinic, compared to women whose nearest clinic remained open [13]. Women who completed the in-depth interviews not only spoke about the types of costs associated with attending multiple visits, including lost wages for themselves and their support network, and paying for child care, gas or public transportation, but also how poverty often compounded the barriers they faced. This led some to consider not having the abortion, and one woman ultimately decided to continue her pregnancy at least in part because of the obstacles she encountered. This adds to other documented cases of women in Texas who have been prevented from having a desired abortion because their nearest clinic closed after HB 2 was enforced [12].

This study also adds to the limited qualitative research describing the range of barriers faced by women who must travel long distances to access abortion services [20]. Beyond financial costs, having to travel longer distances for care imposes social costs for women. Women’s social networks were essential for helping them minimize the out-of-pocket expenses associated with their clinic visits, including borrowing a car or getting a ride and assistance with childcare, a finding also noted among Australian women traveling long distances [21]. However, there were women who expressed fear of stigma by friends and family or ways that drawing on these networks might compromise their desire for confidentiality. For example, being forced to disclose their abortion to people they would not have had to tell if they could have obtained the procedure closer to home, a theme that has also been reported among women traveling long distances for abortion in Alabama, Australia and Scotland [20–22].

Similar to findings from other studies, many women in our sample had a strong preference for medication abortion [23, 24]. Lack of information about the law’s requirements and the rush to find and get to an open clinic before passing the gestational age limit were challenging for most women who wanted this method. Some were able to successfully navigate the long distances and multiple visits needed to realize their preference, while others were not. These findings give insight into the ways in which clinic closures in Texas compounded the difficulties that women experienced in obtaining a desired medication abortion under the new regulations. This is supported by findings from the survey demonstrating that women whose nearest clinic closed after HB 2 was implemented were more likely to report an unmet demand for medication abortion compared to those whose nearest clinic remained open [13]. More research is needed in order to better understand the impact on women of not being able to obtain a desired medication abortion.

It is also important to note that the restrictions on medication abortion may also increase the medical risks of the procedure. Women who traveled long distances to access medication abortion have to cope with starting the abortion process during their travel back home after taking the misoprostol in the clinic, a finding that was also noted among Scottish women traveling long distances for care [22]. Other research has documented an increased risk of needing additional medication or surgical intervention to complete the abortion when the protocol described in the FDA-approved mifepristone label is used compared to other evidence-based protocols [25]. While more research is needed to quantify the risks of required in-clinic administration of misoprostol followed by subsequent travel home for patients, our findings suggest that for some women, the requirements in HB 2 may have made the process of medication abortion more difficult than it would have been in the absence of the law.

It is important to note a few of the limitations of this study. The perspectives presented in this paper do not necessarily address barriers faced by several important groups of women, including those who primarily speak Spanish and those who desire abortion care but are unable to get to the clinic at all. Furthermore, there is the possibility of selection bias given the low participation rate despite multiple attempts to contact all women who were eligible to participate. It is important to note that these data were collected in 2014 and abortion access across the state has continued to change. Additional clinics have closed, the wait times for appointments in some cities have intermittently been very long [26], and some clinics have resumed offering medication abortion. The fact that the FDA updated the mifepristone label in March 2016 appears to have nullified the HB 2’s restrictions on medication abortion. The study’s strengths are that it documents the impact of long travel distances and medication abortion restrictions on women’s access to timely abortion care after three of four provisions of HB 2 were implemented in Texas. It complements other survey data on this topic. The study describes a range of experiences, including one woman who decided to continue the pregnancy. The themes that emerged in these findings may be useful to guide research and advocacy efforts related to similar abortion restrictions in other US states.

Taken together these results provide further insight into the burdens imposed by HB 2 and the ways that the Texas abortion law has limited women’s access to abortion [4, 12, 13]. Abortion is a component of comprehensive reproductive health care, and denying women access to this service is increasing recognized as a breach of human rights [4]. The recent Supreme Court ruling that the two provisions of HB 2 were unconstitutional because of the undue burden on access they created is undoubtedly a victory for women’s rights. However, it will take time before clinics reopen and access is restored for women across the state. Future research will need to explore the long-term impact of HB 2 and similar laws on unwanted births, gestational age at time of abortion and abortion self-induction among women in Texas and elsewhere.
